# A Complete Pronouncing Medical Dictionary

**Published:** 1885-12

**Authors:** 


A COMPLETE PRONOUNCING MEDICAL DICTIONARY. *By* JOSEPH THOMAS, M. D., LL. D. *Philadelphia:* J. B. LIPPINCOTT & CO. *Chicago:* W. T. KEENER.

This is an enlargement and modification of another work by the same author, and is especially intended for the assistance of the large number of medical men who have never enjoyed the advantages of a full training in the ancient languages. Besides being a guide to pronunciation, it is quite full on the etymology of medical terms as far as this can be traced; which is certainly a valuable feature and one which will commend it generally. Yet it cannot be said that it fills its mission fully. J. H. L.

